# Postbiotic muramyl dipeptide alleviates colitis *via* activating autophagy in intestinal epithelial cells

**DOI:** 10.3389/fphar.2022.1052644

**Published:** 2022-11-23

**Authors:** Yaying You, Yongtao Xiao, Ying Lu, Jun Du, Hui Cai, Wei Cai, Weihui Yan

**Affiliations:** ^1^ Division of Pediatric Gastroenterology and Nutrition, Xinhua Hospital Affiliated to Shanghai Jiao Tong University School of Medicine, Shanghai, China; ^2^ Shanghai Institute for Pediatric Research, Shanghai, China; ^3^ Shanghai Key Laboratory of Pediatric Gastroenterology and Nutrition, Shanghai, China

**Keywords:** MDP, intestinal barrier, colitis, NOD2, autophagy

## Abstract

The pathogenesis of IBD is complicated and still unclear. Nucleotide-binding oligomerization domain 2 (NOD2) plays a significant role in regulating gut inflammation under the activation of muramyl dipeptide (MDP), which is used as a postbiotic. The study aimed to investigate the effect of MDP on the intestinal barrier in colitis and the mechanism involved. In this study, C57BL/6 mice were challenged with dextran sodium sulfate (DSS) for establishing a colitis model with the pre-treatment of MDP *in vivo*. Intestinal permeability was reflected by detecting the serum concentration of 4 kDa Fluorescein Isothiocyanate-Dextran. The expression of inflammation, barrier-related proteins, and autophagy was tested by Western Blotting. Proliferation and apoptosis in intestinal epithelial cells were detected by immunohistochemistry. Caco-2 cells were exposed to lipopolysaccharide for imitating inflammation *in vitro*. The findings showed that administration of MDP ameliorated losses of body weight loss, gross injury, and histology score of the colon in the DSS-induced colitis mice. MDP significantly ameliorated the condition of gut permeability, and promoted intestinal barrier repair by increasing the expression of Zonula occludens-1 and E-cadherin. Meanwhile, MDP promoted proliferation and reduced apoptosis of intestinal epithelial cells. In the experiment group treated with MDP, LC3 was upregulated, and p62 was downregulated, respectively. These results suggested that MDP stimulation attenuates intestinal inflammation both *in vivo* and *in vitro*. Potentially, MDP reduced the intestinal barrier damage by regulating autophagy in intestinal epithelial cells. Future trials investigating the effects of MDP-based postbiotics on IBD may be promising.

## 1 Introduction

Muramyl dipeptide (MDP) is a component of bacterial cell walls, and plays an essential role in maintaining intestinal immune balance by activating nucleotide-binding oligomerization domain 2 (*NOD2*), an intracellular pattern recognition receptor (Caruso et al., 2014). The polymorphisms of *NOD2* are investigated as a risk factor for Inflammatory bowel disease (IBD) (Fritz et al., 2011). At present, the role of *NOD2* in IBD has not been clearly elucidated. NOD2 includes three domains: caspase activation and recruitment domains (CARDs), nucleotide-binding oligomerization domain (NOD), and leucine-rich repeat (LRR) (Fritz et al., 2011). When LRR is combined with MDP, CARDs unfold and modulate signal transmission. The variations or defection of *NOD2*, which are most common in frameshift mutations in the receptor LRR region, would cause bacterial invasion, leading to intestinal inflammation and epithelial barrier destruction (Hugot et al., 2001; Al Nabhani et al., 2017). Existing studies have demonstrated that *Nod2* knockout mice displayed more severe enteritis compared with wild-type mice in the experimental colitis model (Watanabe et al., 2008). NOD2 activated by MDP could reduce intestinal injury in murine colitis and MDP may inhibit Toll-like receptors (TLR2 or TLR4) mediated pro-inflammatory signal pathway to decrease the expression of nuclear factor kappa-B (NF-κB) (Watanabe et al., 2008). In addition, MDP could also activate NOD-like receptor 1 (NLR1), which contributes to initiating inflammasome assembly, and participates in the activation of procaspase-1 and then regulating the expression of proinflammatory cytokine interleukin-1β (IL-1β) (Kersse et al., 2011). Otherwise, NOD2 activated by MDP could stimulate NF-κB and mitogen-activated protein kinase (MAPK) signaling pathways, which also modulate the expression of NLR1 in inflammatory environment and play a significant role in innate immune (Kersse et al., 2011; Fann et al., 2018). Besides, postbiotics which include microbial metabolites or their components, have effects on the immunomodulation, anti-inflammation, and antioxidant (Żółkiewicz et al., 2020). The usage of postbiotics for alleviating diseases (such as metabolic diseases or intestinal disorders) has been widely discussed (Li et al., 2021; Mayorgas et al., 2021). MDP as a postbiotic, has been found to improve blood glucose and metabolic inflammation, and the MDP-based drug, like mifamurtide, is applied in therapy (Cavallari et al., 2017). The potential application of MDP-based postbiotics in IBD attracts our attention.

IBD is a chronic and nonspecific inflammatory disorder of the gastrointestinal tract, including Crohn’s disease (CD), ulcerative colitis (UC), and indeterminate colitis (IC) (Yu and Rodriguez, 2017; Emile et al., 2020). The incidence of IBD is increasing, and IBD patients are gradually getting younger (Kuenzig et al., 2022). The etiology of IBD is not elucidated clearly. However, it is commonly considered that genetic and environmental factors perform significant roles in genetically susceptible individuals (Salim and Söderholm, 2011). Obvious damage of intestinal epithelium, which is one of the factors affecting disease recovery, could be investigated in IBD patients and murine colitis model (Muise et al., 2009; Ibrahim et al., 2020). Intestinal epithelial cells (IECs) establish a barrier by the tight junction (TJ), adherens junction, and the desmosome (Buckley and Turner, 2018). These proteins interdict the paracellular pathway and seal the permeability of the entry of toxins and pathogens (Ghosh et al., 2021). It is certified that Zonula occludens-1 (ZO-1) and E-cadherin were downregulated in IBD, followed by the raising of intestinal permeability (Muise et al., 2009). Therefore, it is essential to reduce intestinal injury by maintaining IECs’ proper barrier function in IBD.

In addition, NOD2 has recently been investigated in the function of autophagy induction in inflammatory disorders (such as IBD) (Fritz et al., 2011). Autophagy is a process of swallowing its own cytoplasmic proteins or intracellular pathogens, packing them into vesicles, and fusing them with the lysosome to degrade the contents. As a cellular stress response, autophagy realizes cellular homeostasis and cell survival (Foerster et al., 2022). Autophagy enhances the ability of cells to resist external abnormalities by participating in mediating tight junction regulation and protecting epithelial cells (Nighot et al., 2015; Foerster et al., 2022). The role of NOD2 in autophagy is still under debate. To date, NOD2 is considered as a recruiter of autophagy-related 16-like protein 1 (ATG16L1) to the plasma membrane where the bacteria enter. Interestingly, *ATG16L1* and *NOD2* mutations are both associated with increased susceptibility to CD (Homer et al., 2010).

Based on the above, to evaluate the functional role of MDP during intestinal inflammation, our study investigated the effects of MDP on the dextran sodium sulfate (DSS)-induced colitis model mice and lipopolysaccharide (LPS)-induced inflammation in Caco-2 cells, respectively. Following the detection of the change in intestinal inflammation and gut barrier, this study also found a significant connection between MDP and intestinal barrier protection.

## 2 Material and methods

### 2.1 Ethical statement

All animal experiments were approved by Ethics Committee of Xinhua Hospital Affiliated to Shanghai Jiao Tong University School of Medicine (XHEC-F-2022-057). All procedures were following institutional guidelines for ethical animal studies.

### 2.2 Materials

Primers used in quantitative real-time polymerase chain reaction (qRT-PCR) analysis were synthesized by Invitrogen, including Tubulin and IL-1β. The primers were listed in following: IL-1β-F, 5′- CAG​AAG​TAC​CTG​AGC​TCG​CC- 3′, IL-1β-R, 5′- AGA​TTC​GTA​GCT​GGA​TGC​CG- 3′; Tubulin-F, 5′-CGT​GTT​CGG​CCA​GAG​TGG​TGC- 3′, Tubulin -R, 5′-GGG​TGA​GGG​CAT​GAC​GCT​GAA- 3′. The information of primary antibodies was listed in Table 1. NOD2 agonist MDP (Cat.No.A9519) was obtained from Sigma-Aldrich, LPS from *Escherichia coli* O55:B5 (Cat.No.L6529) from Sigma-Aldrich, DSS (MW 36,000–50,000, Cat. No.160110) from MP Biomedicals, hemoccult fecal occult blood qualitative detection kit (Cat.No.R21607) from Yuanye, TdT-mediated dUTP Nick-End Labeling (TUNEL) Assay Kit (Cat.No.G1507) from Servicebio, and LDH Cytotoxicity Assay Kit (Cat.No.C20301) from Invitrogen.

**TABLE 1:** Antibody information.

Antibody	Source	Catalog#	Application/dilution
ZO-1	Bioss inc.	bs-1329R	WB(1:1000)
E-cadherin	Cell Signaling Technology	3195P	WB(1:1000)
beta-Actin	Cell Signaling Technology	4970S	WB(1:1000)
GAPDH	Lablead	G0100	WB(1:5000)
NOD2	Proteintech	66710-1-Ig	WB(1:1000)
LC3A/B	Cell Signaling Technology	12741S	WB(1:1000)
p62/SQSTM1	R&D	MAB8028	WB(1:500)
Cleaved Caspase-3	Cell Signaling Technology	9664S	WB(1:1000)
TNF-α	Cell Signaling Technology	8184S	WB(1:1000)
Ki67	Servicebio	GB111141	IHC (1:500)

WB: western blotting; IHC: immunohistochemistry.

### 2.3 Animals and cells

Six-week-old male C57BL/6 mice were purchased from Shanghai Jihui Experimental Animal Breeding Company and were adaptively fed for 1 week. Animals were allowed free access to standard laboratory chow and tap water, and the environment was kept on 12-12 h light/dark cycles.

Caco-2 cells were cultured at 37°C in a humidified atmosphere of 5% CO_2_ in Dulbecco modified Eagle medium (Cat. No.10-013-CV, Corning) with 20% fetal bovine serum (Cat.No.10270-106, Gibco), 1% penicillin/streptomycin (100×) (Cat.No.G4003, Servicebio), and 1% Non-Essential Amino Acids Solution (100×) (Cat.No.11140050, Thermo Fisher Scientific).

### 2.4 DSS-induced colitis

C57BL/6 mice were administrated with DSS (3.0% wt/vol) (MW 36,000–50,000, Cat. No.160110, MP Biomedicals) in drinking water for 7 days (Day 1–7), while mice in the control group drank tap water normally. At the early phase of colitis induction, mice were given intraperitoneal injection (i.p.) with MDP (100 μg) or sterile phosphate-buffered solution (PBS) 100 μL on Day 1, 2, and 3, respectively (Watanabe et al., 2008). The mice were euthanized on Day 8: mice were given 1% pentobarbital sodium (7 μL/g body weight, i.p.) to be anesthetized and then be put to death by cervical dislocated after bloodletting.

### 2.5 Disease activity index

The disease activity index (DAI) was determined by evaluating weight loss, stool consistency, and fecal blood in each mouse. A score was adapted from Cooper et al. (Cooper et al., 1993) with minor adjustment. Briefly, the score was defined as follows: Weight loss: 0, none; 1, 1-5% loss; 2, 6-10% loss; 3, 11-18% loss; 4, >18% loss; Stool consistency: 0, normal; 1, soft but still formed; 2, soft; 3, very soft, wet; 4, watery diarrhea; Blood: 0, negative hemoccult; 1, weak positive hemoccult; 2, positive hemoccult; 3, blood traces in stool visible; 4, gross rectal bleeding. Stool blood was detected using the hemoccult fecal occult blood test (Yuanye. Shanghai, China). The total DAI score is given as a sum of the three parameter results.

### 2.6 Intestinal permeability *in vivo*

The day before euthanasia, mice were fasted overnight and given with 4 kDa fluorescein isothiocyanate (FITC)-dextran (Cat. No.46944, Sigma-Aldrich) dissolved in PBS (600 mg kg−^1^ body weight), based on the methodology reported by Hidalgo-Garcia et al. (Hidalgo-Garcia et al., 2021). Four hours later, the mice were anesthetized by 1% pentobarbital sodium (7 μL/g body weight, i. p.). Blood samples were taken from the eyeball and immediately stored in the dark. Serum was separated by centrifuging at 4,000 × g for 10 min at 4°C, and diluted in PBS (1:10). A standard curve was obtained by serial dilutions of FITC-dextran in PBS. Levels of FITC-dextran in the blood were detected by fluorescence spectrophotometry at an excitation wavelength of 485 nm and emission wavelength of 520 nm.

### 2.7 Histological analysis

Terminal ileum and colon were harvested on Day 8. Colon and terminal ileum tissues were stained with hematoxylin and eosin (H&E) following the protocol (Servicebio, Wuhan, China). Histological analysis method was adapted from Wu et al. (Wu et al., 2020) with minor adjustments. Briefly, histology was scored for murine colitis from inflammatory infiltration (0-5, submucosal edema (0-5), and the severity of ulceration (0-5). The total maximum score is 15. Histological images were collected with three visual fields per sample, and the mean score was recorded. In addition, nine villi and crypts of the terminal ileum in each sample were measured, and the mean value was recorded.

### 2.8 Assessment of apoptosis by TUNEL

Terminal ileum and colon were fixed by 4% paraformaldehyde (PFA) and embedded in paraffin. Apoptosis was performed using TUNEL staining according to the manufacturer’s instructions (Servicebio, Wuhan, China). Briefly, intestine sections were deparaffined and rehydrated using xylene and gradient descending ethanol. Antigen retrieval was performed using proteinase K (Cat.No.G1205, Servicebio) working solution incubated at 37°C for 20 min, followed by immersing in 3% H_2_O_2_ at room temperature (RT) for 20 min from light to block endogenous peroxidase. After keeping the equilibrium at RT in the buffer, the slices mixed with Tunel reaction solution (Recombinant TdT enzyme: Biotin-dUTP Labeling Mix: Equilibration Buffer = 1 µL: 5 µL: 50 µL) for 1 h and streptavidin-HRP for 30min in a flat wet box at 37°C, respectively. Subsequently, positive signals were visualized using a diaminobenzidine (DAB) kit (Cat.No.G1212, Servicebio) and counterstained with Hematoxylin staining solution. Normal nuclei stained with hematoxylin were blue, while the positive apoptosis cells developed by the DAB reagent had brown-yellow nuclei and were counted. Five visual fields were randomly selected from each sample. Positive apoptosis cells and the area of intestinal epithelium were quantified, and the apoptosis index was presented as the positive apoptosis cell number per mm^2^ observed by CaseViewer (3DHISTECH, Budapest, Hungary).

### 2.9 Immunohistochemistry

Immunohistochemistry (IHC) was presented using the DAB reagent following the protocol (Servicebio, Wuhan, China). Briefly, paraffin-embedded intestinal tissues were deparaffinized and rehydrated. Citrate antigen retrieval solution (pH 6.0) was used for antigen retrieval. Endogenous peroxidase was blocked using 3% H_2_O_2_ and then immersed in 10% bovine serum albumin. Slides were incubated with the primary antibody, Ki67 (Servicebio, dilution, 1: 500), overnight at 4°C in a wet chamber and secondary antibody at RT for 50 min, respectively. Positive cells were marked using DAB chromogenic reagent and counted. The IHC was analyzed by CaseViewer (3DHISTECH, Budapest, Hungary) with 5 fields per sample.

### 2.10 Caco-2 cell treatment

Caco-2 cells were seeded into 12 well-plates in a concentration of 4 × 10^5^ cells per well and incubated at 37°C with 5% CO_2_ for 24 h. To choose an appropriate concentration of MDP, cells were treated with MDP (0, 1, 5, or 10 μg/ml) for 0.5 h, and then total proteins were extracted.

To choose an appropriate concentration of LPS, Caco-2 cells were incubated with LPS (0, 1, 10, or 100 μg/ml) for 24 h, respectively. Cytotoxicity was tested, and cellular RNA was extracted.

Caco-2 cells were seeded into 12 well plates and cultured for 24 h. Caco-2 cells were then pre-treated with MDP for 0.5 h or not, followed by the stimulation of LPS for 24 h. Control groups were incubated in a regular medium for 24 h. After that, the supernatant was removed, and cell proteins were extracted for further investigation.

### 2.11 Cytotoxicity test

Cellular cytotoxicity was quantified using a commercially LDH Cytotoxicity Assay Kit following the manufacturer’s guidelines (Invitrogen, Carlsbad, CA). Caco-2 cells (1 × 10^4^ cells) were plated on a 96-well plate and cultured overnight. The supernatant (50 µL/well) was transferred into a new 96-well plate, and a 50 µL reaction mixture was added to each well. The plate was incubated for 30 min at RT from light. Subsequently, 50 µL Stop Solution was added to each sample well to terminate the reaction. The absorbance was measured at 490 nm and 680 nm (background).

### 2.12 Western blotting analysis

Tissues and Caco-2 cells were lysed in RIPA lysis buffer (Cat.No.G2002, Servicebio) on ice. Protein concentrations were determined by the bicinchoninic acid (BCA) protein assay kit (Beyotime Inst. Biotech, Beijing, China). Samples with equivalent amounts of total protein (30 μg) were loaded and separated on NuPAGE 10% Bis-Tris gels (Invitrogen, Carlsbad, CA) and transferred to polyvinylidene difluoride (PVDF) membranes. The membranes were blocked in 5% nonfat milk for 1.5 h at RT and were then incubated with the specific primary antibodies overnight at 4°C. After washing three times for 30 min with Tris Buffered Saline with Tween (TBST), the membranes were incubated with HRP-conjugated secondary antibody for 1 h at RT. Subsequently, washing with TBST again, the blots were visualized with an ECL chemiluminescence reagent kit (Pierce, Rockford, IL, United States) and quantified by ImageJ (NIH, Bethesda, Maryland, United States). The primary antibodies were shown in Table 1. The secondary antibodies included Anti-rabbit Antibody (Cat. No.7074, Cell Signaling Technology) and Anti-mouse Antibody (Cat. No.7076, Cell Signaling Technology).

### 2.13 qRT-PCR analysis

Total RNA was extracted from Caco-2 cells using RNAsimple Total RNA Kit (TIANGEN, Beijing, China) according to the manufacturer’s instructions. RNA concentration was determined using Nano-drop spectroscopy (Applied Biosystems, Foster City, CA). Reverse transcriptions were finished by The High Capacity cDNA Reverse Transcription Kit (Applied Biosystems, Foster City, CA). The complementary DNA was analyzed by RT-PCR using the PowerUp SYBR-Green Master Mix kit (Applied Biosystems, Foster City, CA) by the ViiA 7 Real-Time PCR System (Applied Biosystems, Foster City, CA) with the program of 95°C for 10 min, following 40 cycles at 95°C for 15 s and 60°C for 1 min. All gene expression results were normalized to endogenous controls as Tubulin expression. The ^ΔΔ^Ct method was employed to analyze the relative RNA expression levels.

### 2.14 Statistics

All results were presented as the mean ± SEM. For comparisons of different groups, statistical significance was determined based on One-way or Two-way ANOVA analysis. *p* values < 0.05 were considered to be statistically significant. All analyses were conducted with GraphPad software (version 9; GraphPad Software Inc., San Diego, CA).

## 3 Results

### 3.1 MDP alleviates DSS-induced intestinal injury in C57BL/6 mice

**FIGURE 1:**
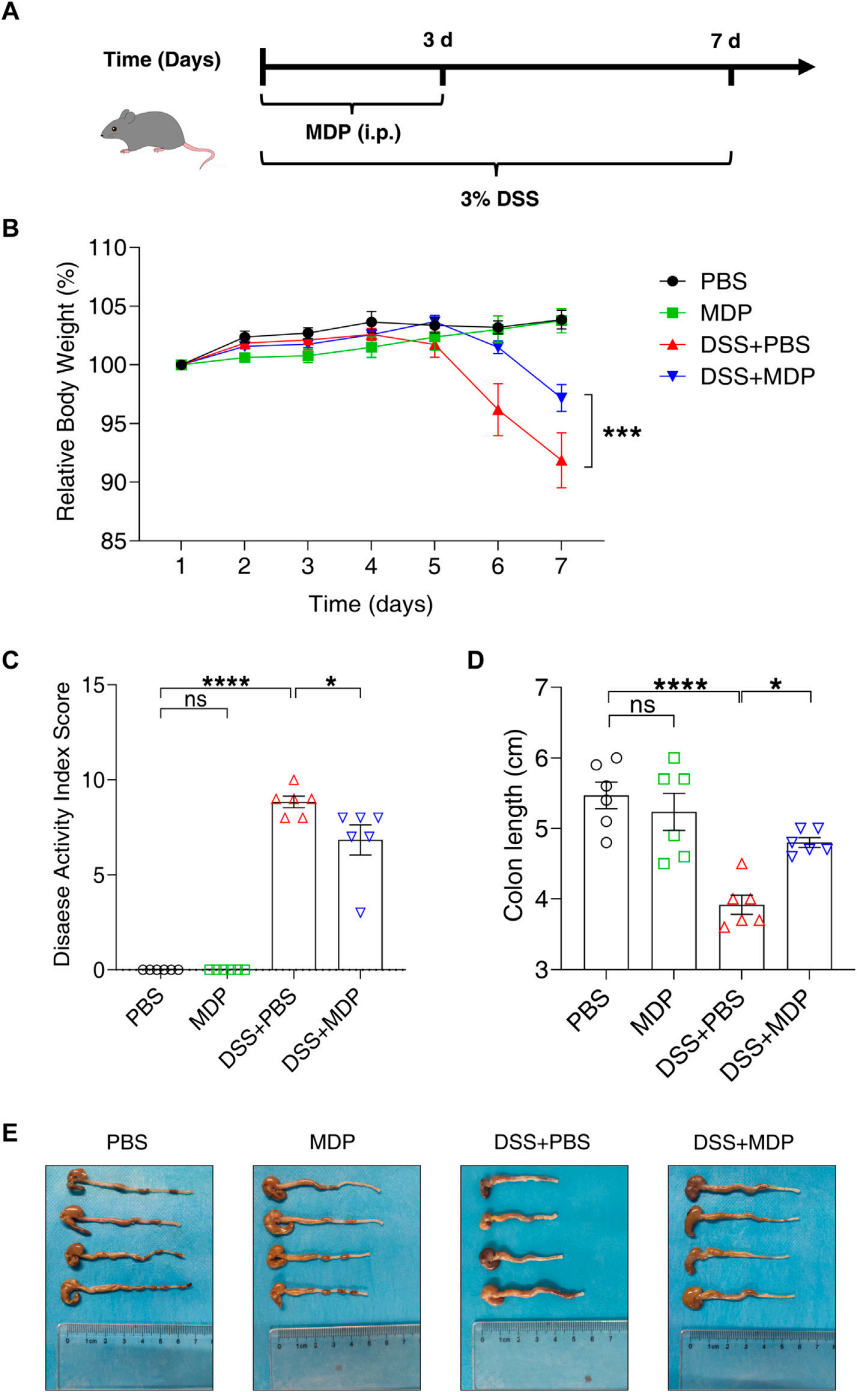
MDP alleviates DSS-induced intestinal injury in C57BL/6 mice. **(A)** Experimental timeline was shown. C57BL/6 mice were divided into four groups: PBS group, MDP group, DSS + PBS group, and DSS + MDP group (n = 6, each group). Each group received continuous intraperitoneal injections (i.p.) of MDP (100 μg) or sterile PBS 100 μL from Day 1 to Day 3, respectively. The mice drank water or 3% DSS from Day 1 to Day 7. **(B)** Body weight of mice was recorded every day. **(C)** Weight loss, stool consistency, and blood of mice were observed for evaluating disease activity index scores. **(D,E)** Mice were euthanized on Day 8. The colon was taken and the length was measured and photographed. Data were expressed as mean ± SEM. *p <0.05, ***p <0.001, ****p <0.0001, ns, not significant, One-way or Two-way ANOVA.

To evaluate the protective effect of MDP in colitis, C57BL/6 mice were divided into four groups: PBS group, MDP group, DSS + PBS group, and DSS + MDP group (n = 6, each group). We fed mice with DSS (3.0% wt/vol) in drinking water in experiment groups for 7 days to construct the colitis model (Figure 1A). Each group was administrated with MDP (100 μg) or PBS intraperitoneal injection (i.p.) for 3 consecutive days (Day 1 to Day 3). Compared with mice given PBS, MDP-treated mice displayed significantly less weight loss during the process of DSS-induced colitis (Figure 1B). The Disease Activity Index (DAI) of the DSS + MDP group was lower compared with DSS + PBS group (Figure 1C). The length and gross injury of the colon were observed in detail (Figures 1D,E). DSS + PBS group showed colon length significantly shortened, the cecum shrunk, and obvious bleeding spots could be seen on the colon which manifested with hyperemia, edema, and embrittlement. These negative impacts were ameliorated by MDP administration (Figures 1D,E).

**FIGURE 2:**
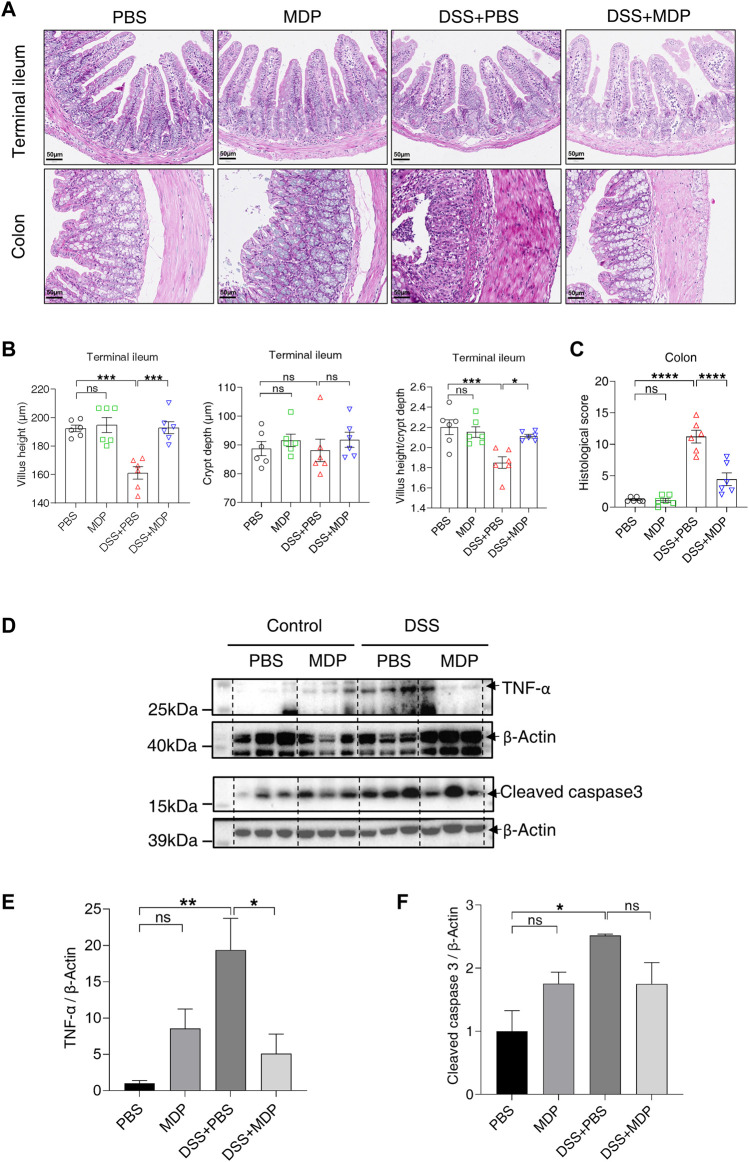
MDP inhibits inflammatory response in the intestine. **(A)** Representative images of H&E staining for terminal ileum and colon tissues in PBS, MDP, DSS + PBS, and DSS + MDP group mice (n = 6, each group), respectively. scale bars: 50 μm. **(B)** Quantification of villus height, crypt depth, and the ratio of villus height and crypt depth in the terminal ileum (n = 6, each group). **(C)** The histological score of colons (n = 6, each group). **(D)** Western blotting analysis for TNF-α and Cleaved caspase 3 with β-Actin as the internal standard protein in colon of PBS, MDP, DSS + PBS, and DSS + MDP group mice. Representative images of three duplicate samples of the immune blotting were shown. **(E,F)** Quantification of the relative expression of TNF-α and Cleaved caspase 3 in panel (D) by ImageJ, respectively. Data were expressed as mean ± SEM. *p <0.05, **p <0.01, ***p <0.001, ****p <0.0001, ns, not significant, One-way ANOVA.

To further explore the effect of MDP on intestinal inflammation induced by DSS *in vivo*, pathological changes were evaluated by H&E in the terminal ileum and colon, respectively (Figure 2A). DSS-induced mice with PBS (i.p.) displayed shorter villus height, and a lower ratio of villus height and crypt depth in the terminal ileum, but not in the mice of the DSS + MDP group (Figure 2B). In the DSS + PBS group, H&E staining showed that colon tissues had large ulcers in mucosa with the disappearance of gland. And the inflammatory reaction was severe, neutrophil infiltration could be seen in mucosa, submucosa and even muscle layer (Figure 2A). Compared with the DSS + PBS group, the DSS + MDP group successfully improved the histological scores of colons (Figure 2C). The Western blotting analysis demonstrated that DSS induced the high expression levels of TNF-α and cleaved caspase 3 in the colon tissues compared with the control group (Figures 2D–F). Compared with the DSS + PBS group, TNF-α was downregulated in the DSS + MDP group (Figure 2E).

### 3.2 MDP reduces DSS-induced intestinal barrier damage

**FIGURE 3:**
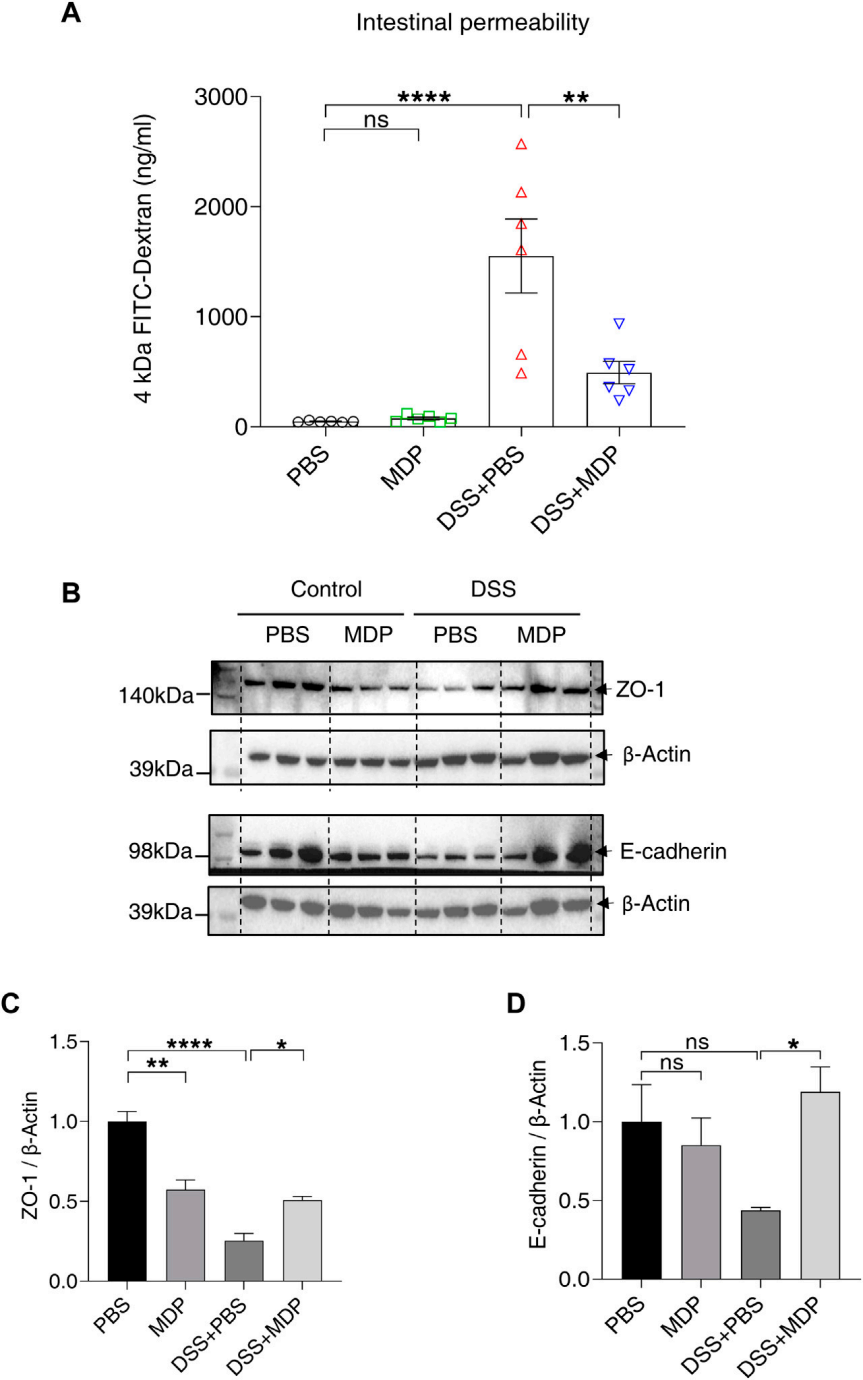
MDP reduces DSS-induced intestinal barrier damage. **(A)** Mice were fasted overnight on Day 7 and given FITC-Dextran by gavage on Day 8. After 4 h, blood was taken from the eyeball from light. The serum was separated and analyzed for the detection of intestinal permeability (n = 6, each group). **(B)** Western blotting analysis for ZO-1 and E-cadherin with β-Actin as the internal standard protein in the colon of PBS, MDP, DSS + PBS, and DSS + MDP group mice. Representative images of three duplicate samples of the immune blotting were shown. **(C,D)** Quantification of the relative expression of ZO-1 and E-cadherin in panel (B) by ImageJ, respectively. Data were expressed as mean ± SEM. *p <0.05, **p <0.01, ****p <0.0001, ns, not significant, One-way ANOVA.

Next, we aimed to study the effect of MDP on intestinal permeability. Mice were given with 4 kDa FITC-dextran by gavage on Day 8, and blood samples were collected for detection. The concentration of FITC-dextran reflected the change of intestinal permeability. Mice in the DSS + PBS group demonstrated higher serum concentrations of FITC-Dextran compared with the control group; while the DSS + MDP group only had a mild change of intestinal permeability compared with the DSS + PBS group (Figure 3A). Furthermore, the Western blotting results revealed that MDP led to higher expression levels of the gut barrier associated proteins, including ZO-1 and E-cadherin, which were significantly downregulated in colon tissues of DSS-induced mice (Figures 3B–D, Supplementary Figure S1).

### 3.3 MDP decreases the apoptosis of intestinal epithelium in DSS-induced mice

**FIGURE 4:**
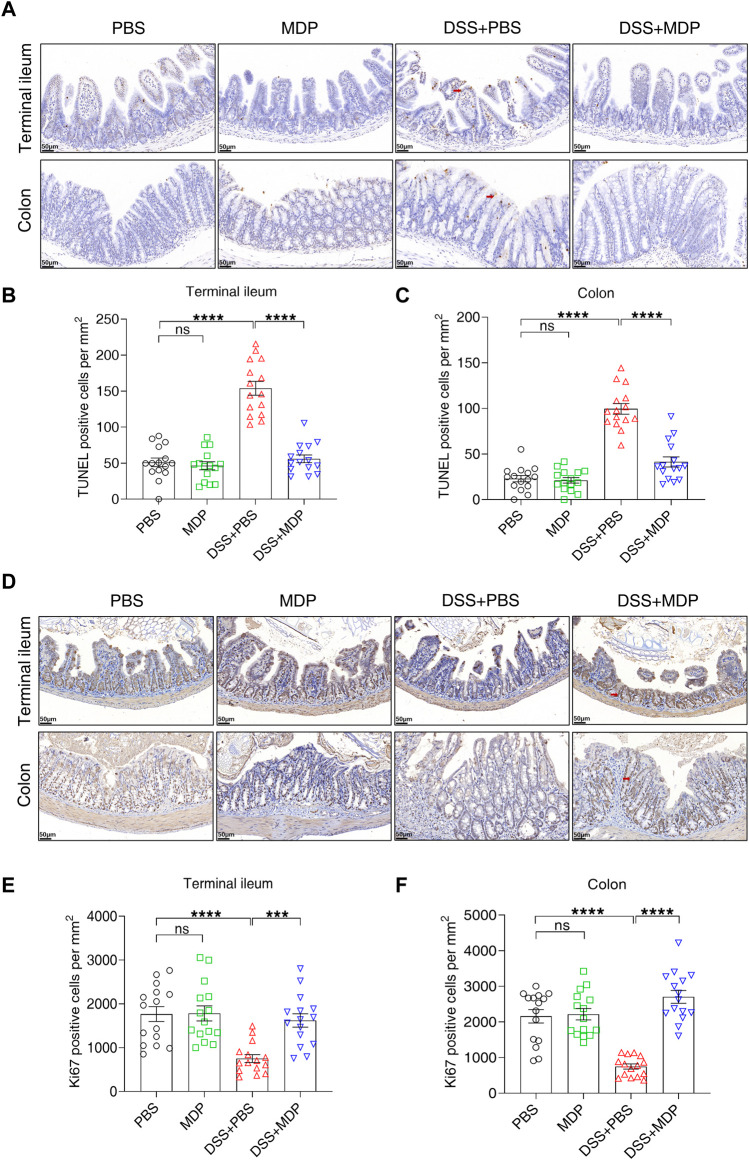
MDP decreases the apoptosis of intestinal epithelium in DSS-induced mice. **(A)** Representative images of TUNEL staining for terminal ileum and colon tissues in PBS, MDP, DSS + PBS, and DSS + MDP group mice (n = 3, each group), respectively. scale bars: 50 μm. Red arrows indicated positive cells. **(B,C)** Quantification of TUNEL positive cells per mm^2^ in terminal ileum and colon (n = 3, each group), respectively. **(D)** Representative images of Ki67 staining for terminal ileum and colon tissues in PBS, MDP, DSS + PBS, and DSS + MDP group mice (n = 3, each group), respectively. scale bars: 50 μm. Red arrows indicated positive cells. **(E,F)** Quantification of Ki67 positive cells per mm^2^ in terminal ileum and colon (n = 3, each group), respectively. Data were expressed as mean ± SEM. ***p <0.001, ****p <0.0001, ns, not significant, One-way ANOVA.

To further investigate the underlying mechanisms of MDP in colitis, the apoptosis (using TUNEL assay) and proliferation (using Ki67 IHC) of IECs were evaluated, respectively (Figure 4). A significant decrease in the number of apoptotic cells in the DSS + MDP group was displayed in comparison to the DSS + PBS group both in terminal ileum and colon (Figures 4B,C). Of note, the quantity of Ki67 positive cells in the DSS + MDP group significantly increased compared with the DSS + PBS group both in terminal ileum and colon (Figures 4E,F).

### 3.4 MDP activates autophagy in the colon of DSS-induced mice

**FIGURE 5:**
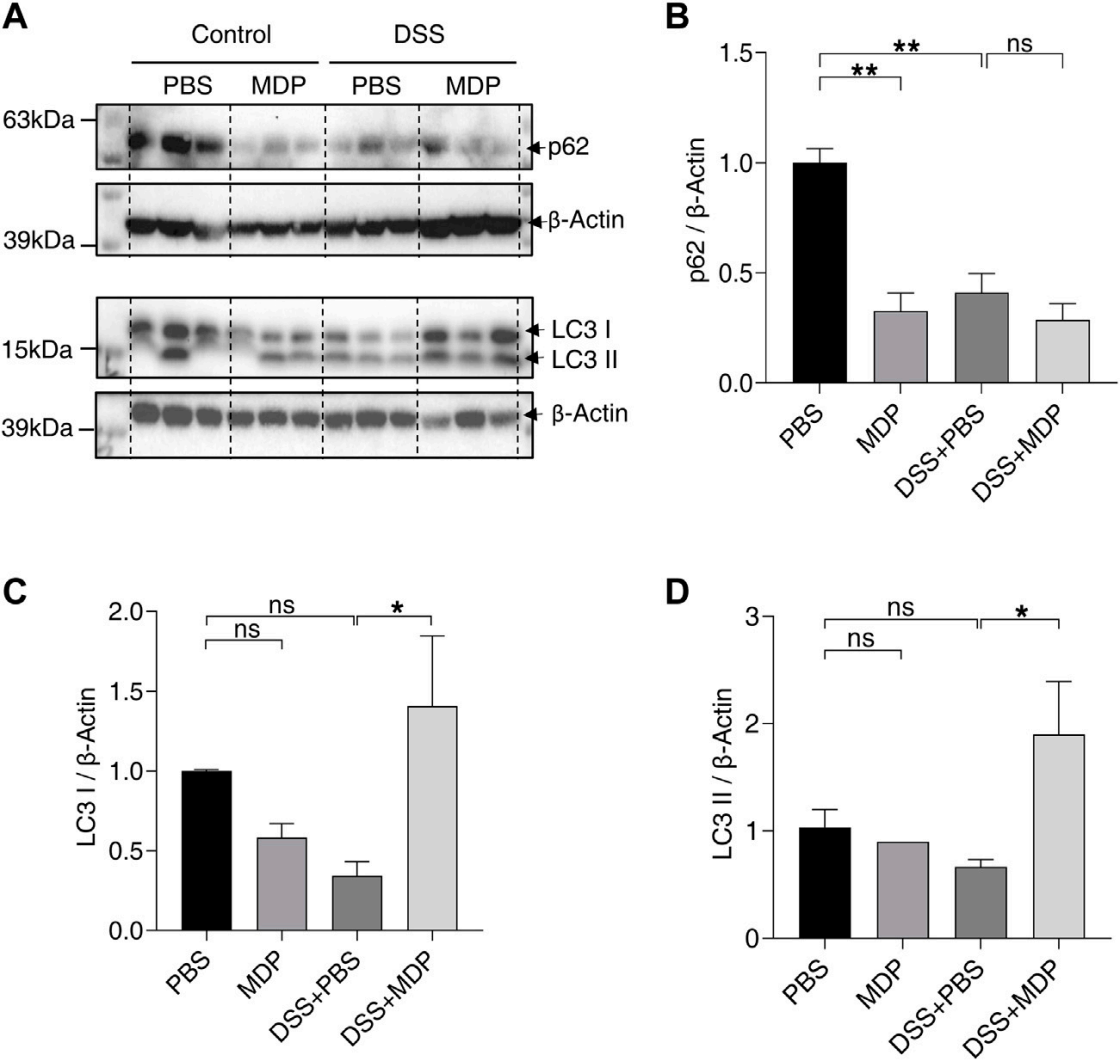
MDP activates autophagy in colon of DSS-induced mice. **(A)** Western blotting analysis for p62, LC3 І, and LC3 ІІ with β-Actin as the internal standard protein in the colon of PBS, MDP, DSS + PBS, and DSS + MDP group mice. Representative images of three duplicate samples of the immune blotting were shown. **(B–D)** Quantification of the relative expression of p62, LC3 І and LC3 ІІ in panel **(A)** by ImageJ, respectively. Data were expressed as mean ± SEM. *p <0.05, **p <0.01, ns, not significant, One-way ANOVA.

As a common intracellular recognition receptor, NOD2 mediates multiple pathways. To determine the relationship between the activation of NOD2 and autophagy, we detected the protein expression levels of p62 and LC3 by Western blotting in colon mucosa tissues (Figure 5A). The expression levels of p62 in MDP, DSS + PBS, and DSS + MDP groups obviously descended in comparison to the PBS group (Figure 5B). The expression levels of LC3 І and LC3 ІІ in the DSS + MDP group both significantly ascended compared with the DSS + PBS group (Figures 5C,D).

### 3.5 MDP reduces intestinal barrier damage and activates autophagy *in vitro*

**FIGURE 6:**
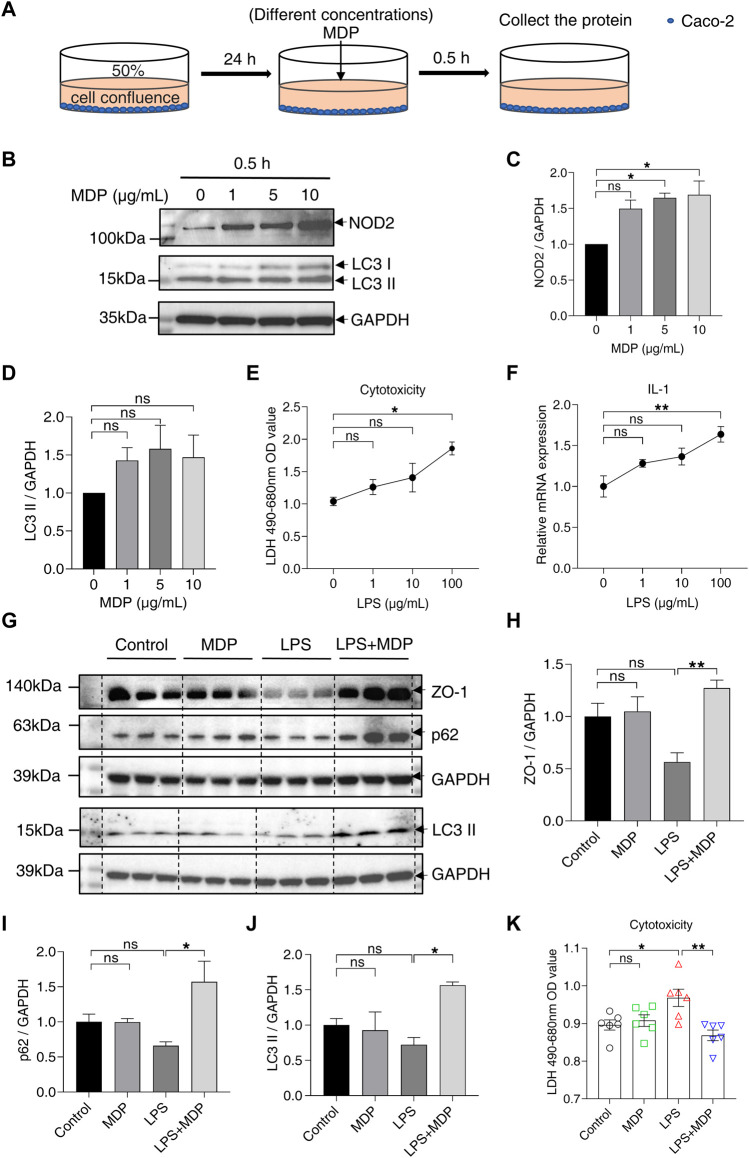
MDP reduces intestinal barrier damage and activates autophagy *in vitro*. **(A)** Schematic diagram of the administration of MDP (0, 1, 5, 10 μg/ml, respectively) in Caco-2 cells. **(B)** Western blotting analysis for NOD2, LC3 І, and LC3 ІІ with GAPDH as the internal standard protein in Caco-2 cells. Representative images of the immune blotting were shown. **(C,D)** Quantification of the relative expression of NOD2 and LC3 ІІ in panel (B) by ImageJ, respectively. Results were from three independent experiments. **(E)** Cytotoxicity of Caco-2 cells with the administration of with LPS (0, 1, 10, 100 μg/mL, respectively) for 24 h by testing LDH OD value. **(F)** Relative RNA levels of IL-1β of Caco-2 cells with the administration of LPS. **(G)** Western blotting analysis for ZO-1, p62, and LC3 ІІ with GAPDH as the internal standard protein in Caco-2 cells of the groups (Control, MDP, LPS, and LPS + MDP), respectively. Representative images of three duplicate samples of the immune blotting were shown. **(H–J)** Quantification of the relative expression of ZO-1, p62, and LC3 ІІ in panel (G) by ImageJ, respectively. **(K)** Cytotoxicity of Caco-2 cells of the groups (Control, MDP, LPS, and LPS + MDP) by testing LDH OD value. Data were expressed as mean ± SEM. *p <0.05, **p <0.01, ns, not significant, One-way ANOVA.

After demonstrating that MDP alleviated inflammation and intestinal barrier damage in the mice model of colitis, we next explored the effect of MDP *in vitro*. Caco-2 cells were stimulated with MDP (gradient increasing concentrations) for 0.5 h (Figure 6A). The Western blotting analysis presented that NOD2 was significantly upregulated in the stimulation of MDP in 5–10 μg/ml (Figures 6B,C). Interestingly, LC3 protein expression displayed a similar trend with NOD2, although there was no statistical difference (Figures 6B,D). Subsequently, Caco-2 cells were treated with LPS to mimic the intestinal inflammation model *in vitro*. The concentration of LPS was chosen by testing cytotoxicity (Figure 6E) and relative mRNA expression of IL-1β (Figure 6F). Results showed that LPS in 100 μg/ml was an appropriate concentration to induce inflammation. The Western blotting results found cells treated with LPS displayed a significant decrease in the expression levels of ZO-1, p62, and LC3 ІІ, but not in the LPS + MDP group (Figures 6G–J). LDH assay test displayed that MDP could significantly reduce cytotoxicity induced by LPS in Caco-2 cells (Figure 6K).

## 5 Discussion

The incidence of IBD is increasing, while the pathogenesis of IBD is ambiguous which may limit the progress of novel treatment. In IBD, intestinal barrier dysfunction is a pathogenic factor which leads to increased intestinal permeability, following immune imbalance between the host and gut microbiota (Salim and Söderholm, 2011). Extensive investigations have indicated that intestinal micro-environmental factors, especially the gut microbiota and their metabolites or components, affect the occurrence and development of IBD (Adak and Khan, 2019; Caruso et al., 2020). MDP is bacterial cell wall-derived, and maintains intestinal homeostasis through NOD2 activation. For instance, MDP inhibits the IL-1β signaling pathway induced by *Pseudomonas fluorescens* (Cruickshank et al., 2008; Alnabhani et al., 2015). The protective mechanism of MDP in IBD is still under exploration. We indicated that MDP mitigates intestinal injury and mechanical barrier damage. Our study showed a new perspective of the function and mechanism of NOD2 and provided potential ideas of postbiotic MDP for the treatment of IBD.

In our research, the intestinal injury was significantly reduced with the administration of MDP in DSS-induced mice by observing the change of body weight, DAI, as well as gross appearance and histology of the colon. Similar results were also detected by Warren Strober et al., and they thought the therapeutic use of MDP is possible (Watanabe et al., 2008). However, Warren Strober further explored the mechanism of MDP in dendritic cells, not IECs. The influence of MDP on intestinal epithelial barrier remains explored. It is known that IBD is associated with intestinal barrier damage, induced by the underlying mechanisms of tight junction abnormality, and apoptotic leaks within the epithelium (Schulzke et al., 2006). In our study, the results indicated MDP upregulates the expression of ZO-1 and E-cadherin in IECs to reduce intestinal permeability, which is significantly decreased in the DSS induced colitis. Our findings also suggest that MDP increases proliferation and decreases apoptosis in intestinal epithelium simultaneously, which explains the function of NOD2 activation in regulating intestinal barrier integrity and protecting against DSS-induced tissue damage.

Anti-TNF-α drugs have been widely applied in clinical treatment and TNF-α is also an important indicator of disease severity (Aardoom et al., 2019; Jongsma et al., 2020). Existing studies found that intestinal inflammation, especially the increase of TNF-α could induce intestinal epithelial tight junction dysfunction (Muise et al., 2009; Ramos and Papadakis, 2019; Ibrahim et al., 2020). Our study found that TNF-α was downregulated under the modulation of MDP in DSS-induced mice. Therefore, we presumed that MDP may reduce the intestinal injury, and increase the expression of ZO-1 and E-cadherin to alleviate gut barrier damage by inhibiting the expression of the pro-inflammatory cytokine. This hypothesis remains to be further tested.

Our findings also suggest that MDP increases proliferation and decreases apoptosis in intestinal epithelium simultaneously, which explains the function of NOD2 activation in regulating intestinal barrier integrity and protecting against DSS-induced tissue damage. Muhammed A Saad et al. (Arab et al., 2021) found the activation of autophagy and suppression of apoptosis concurrently in the course of reducing rat colitis by dapagliflozin. The role of autophagy in the intestinal barrier has been continuously discovered in colitis (Xiong et al., 2019; Foerster et al., 2022). The expression of ZO-1 was found to increase by upregulating autophagy to reduce small intestine injury (Chen et al., 2021). Besides, autophagy deficiency could affect the progression of IBD, and everolimus (an autophagy inducer) has been found to ameliorate CD symptoms (Dumortier et al., 2008). The term “autophagic flux” is used to denote overall autophagic degradation (Zhang et al., 2013). Reduced p62 and increased LC3 ІІ are widely considered to be markers for monitoring autophagic flux when autophagy is activated. The testing of the protein expression of p62 and LC3 in our study suggested that MDP could activate autophagy in the colon of DSS-induced mice. Thus, we could consider that the activation of NOD2 can reduce inflammation by activating autophagy, to improve the intestinal barrier and resist intestinal injury in DSS-induced colitis mice, which also got strong support *in vitro*. Interestingly, the MDP effects on PBS-treated mice may be not consistent with its functions on DSS-treated group, including the autophagy and ZO-1 expression (Figures 3, 5, and Supplementary Figure S1). We speculate that MDP performs an obvious protective effect under the stress state, but NOD2 activation under the balanced physiological environment in healthy mice may be different. Levy et al. (Levy et al., 2020) also showed the similar result. Otherwise, p62 in LPS + MDP group was expressed higher than cells only treated with LPS. This trend could be considered that cells were only stimulated for 24 h, and the degradation procedure of p62 has not started yet. Previous studies have found that activating autophagy mediated by different reagents could alleviate the inflammation responses of murine colitis *in vitro* and *in vivo* (Xiong et al., 2019; Arab et al., 2021), and our research also verified it. Furthermore, dysbiosis in gut microbiota found in the bowel disorder attracts much attention. Novel therapies based on microbiota modulation are promising like postbiotics, prebiotics, and probiotics (Żółkiewicz et al., 2020). Microbial components and their metabolites have been continuously studied and applied to intestinal diseases, such as inositol-1,4,5-trisphosphate and short-chain fatty acids (SCFAs) (Wu et al., 2020; Mayorgas et al., 2021). It may be a promising target to treat gut disorders that the metabolites produced by gut microbiota reduce inflammation of the host by autophagy, although the interplay between gut microbiota and autophagy is still under the exploration.

In conclusion, our results demonstrated a protective effect of MDP with a probable mechanism of autophagy on inflammatory models both *in vivo* and *in vitro*. Our data indicated that MDP enhances the intestinal TJ barrier by increasing the expression of ZO-1, promoting proliferation and reducing apoptosis, and inhibiting the increase of TNF-α. The autophagy signaling pathway may be a relatively novel perspective to explain the protective effect of MDP on inflammation models. The mechanism of NOD2 in intestinal epithelium in the development of colitis still needs to be explored to lay the foundation for MDP-based postbiotics in IBD treatment.

## Data Availability

The original contributions presented in the study are included in the article/Supplementary Material, further inquiries can be directed to the corresponding authors.
