# Supplementary material for: Postbiotic muramyl dipeptide alleviates colitis via activating autophagy in intestinal epithelial cells
Source: Front Pharmacol. 2022 Nov 23;13:1052644. doi: 10.3389/fphar.2022.1052644 (PMC9727138; doi:10.3389/fphar.2022.1052644)
Supplement: Supplementary file 1 [file Image1.pdf]

Supplementary Material

1.1 Supplementary Figures

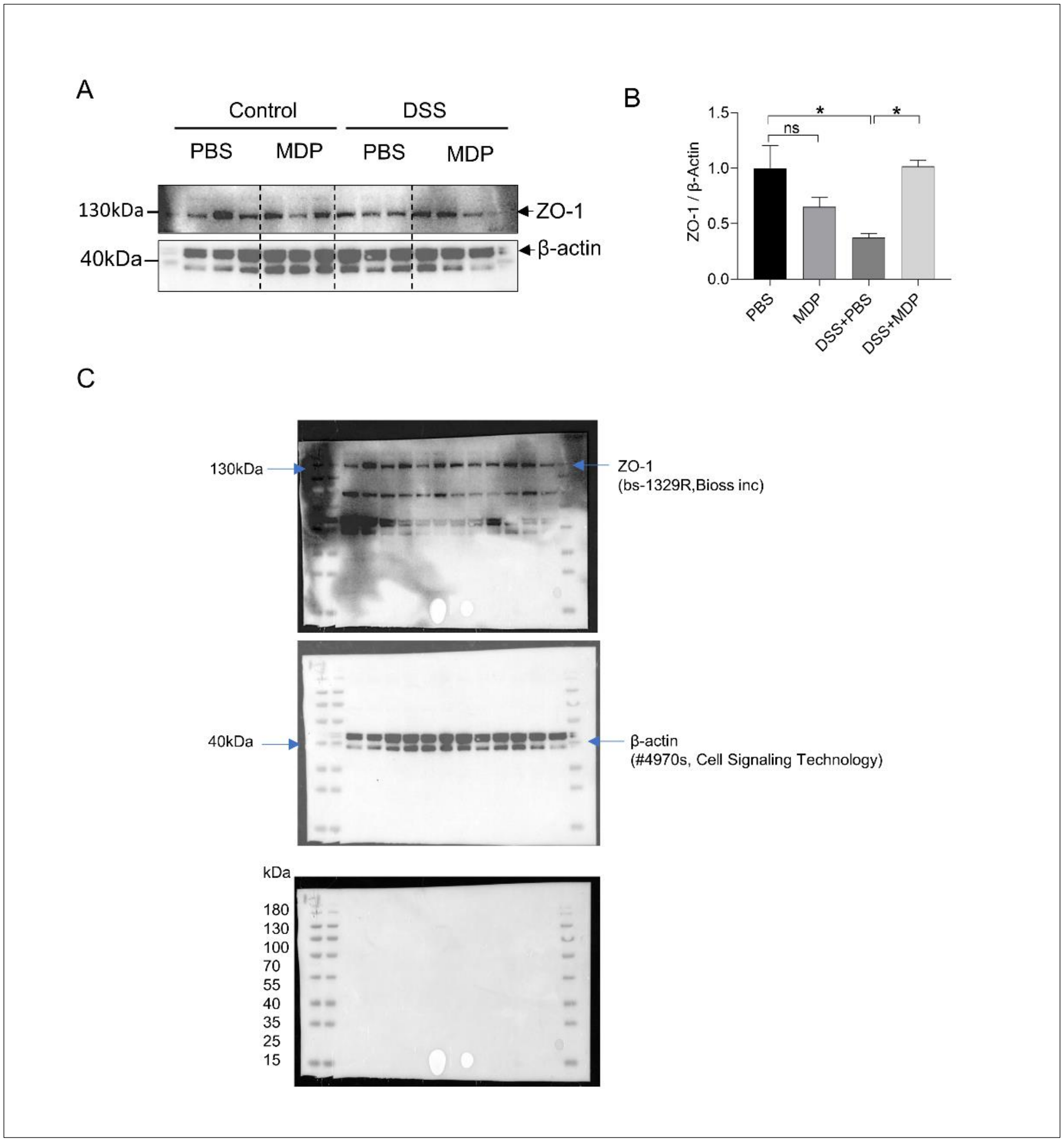

Supplementary Figure 1. MDP changes the expression of ZO-1 in mice. (A) Western blotting analysis for ZO-1 with β-Actin as the internal standard protein in the colon of PBS, MDP, DSS + PBS, and DSS + MDP group mice. Representative images of three duplicate samples of the immune blotting were shown. **(B)** Quantification of the relative expression of ZO-1 in panel (A) by ImageJ,

respectively. **(C, D)** Primary images of panel (A). Data were expressed as mean ± SEM. *P <0.05, ns, not significant, One-way ANOVA.
